# Future of Low-Dose Computed Tomography and Dual-Energy Computed Tomography in Axial Spondyloarthritis

**DOI:** 10.1007/s11926-022-01075-5

**Published:** 2022-04-09

**Authors:** Torsten Diekhoff, Kay Geert A. Hermann, Robert G. Lambert

**Affiliations:** 1 Department of Radiology (CCM), Charité - Universitätsmedizin Berlin, Campus Mitte, Humboldt-Universität Zu Berlin, Freie Universität Berlin, Charitéplatz 1, 10117 Berlin, Germany; 2 Department of Radiology and Diagnostic Imaging, University of Alberta, Edmonton, AB Canada

**Keywords:** Computed tomography, Low-dose computed tomography, Sacroiliac joint, Spine, Sacroiliitis, Spondyloarthritis

## Abstract

**Purpose of Review:**

Recent technical advances in computed tomography (CT) such as low-dose CT and dual-energy techniques open new applications for this imaging modality in clinical practice and for research purposes. This article will discuss the latest innovations and give a perspective on future developments.

**Recent Findings:**

Low-dose CT has increasingly been used for assessing structural changes at the sacroiliac joints and the spine. It has developed into a method with similar or even lower radiation exposure than radiography while outperforming radiography for lesion detection. Despite being incompatible with low-dose scanning, some studies have shown that dual-energy CT can provide additional information that is otherwise only assessable with magnetic resonance imaging (MRI). However, it is unclear whether this additional information is reliable enough and if it would justify the additional radiation exposure, i.e. whether the performance of dual-energy CT is close enough to MRI to replace it in clinical practice.

**Summary:**

While the role of dual-energy CT in patients with axial spondyloarthritis remains to be established, low-dose CT has developed to an appropriate modality that should replace radiography in many circumstances and might supplement MRI.

## Introduction

Computed tomography (CT) has established itself as one of the primary imaging modalities in radiology. Since the invention of the first commercially available scanner in 1972 by Godfrey Hounsfield, it has undergone many developments of hardware, scanning technique and postprocessing to improve the speed of image acquisition and the image quality and reduce the radiation dose [1]. The latest technical advances include the introduction of dual-energy CT (DECT) [2], a term which for this article consists of different methods of spectral CT imaging and iterative and artificial intelligence (AI)-based reconstructions [3]. Both techniques make CT more useful and feasible in rheumatology in general and axial spondyloarthritis (axSpA) in particular. In the following article, discussion will focus on the application of the above techniques to the field of axial spondyloarthritis.

## Comparing CT and MRI

### CT Is Superior to MRI

While radiography and CT are based on the same physical principles, i.e. the attenuation of X-ray photons in tissues, magnetic resonance imaging (MRI) utilizes excitation and relaxation of protons in a magnetic field and, therefore, depicts tissues entirely differently. In conventional MRI sequences — such as T1w, T2w and STIR — signal is generated within fat and water molecules, which are abundant in the bone marrow but sparse within the calcified bone [4]. Therefore, the cortex and trabeculae appear black and are only indirectly visualized on MRI. As a result, subtle changes in those tissues may go unnoticed. On the other hand, CT is very sensitive for the detection of calcification and ossification and can directly visualize bone and, thus, erosion of an articular surface. Although MRI is superior to radiography for erosion detection [5, 6], CT remains the gold standard because it is not restricted to indirect depiction of the bony surface.

Furthermore, the spatial resolution and image quality in MRI are directly linked to the time needed to acquire the images. For MRI, more time = more signal = better images. Therefore, the radiologist must balance image detail, quality and examination time. That is not the case for CT examinations that usually need only a few seconds for image acquisition. With CT, acquisition times are very similar regardless of technique, and it is ‘more radiation exposure’ that = more signal = better images. Therefore, examination time is not a limiting factor for CT but it is for MRI, and radiation exposure is a limiting factor for CT but not for MRI. Moreover, some contraindications to MRI scanning, e.g. claustrophobia or some metal devices, do not apply for CT.

### CT Is Inferior to MRI

CT is a particularly suitable method for depicting structural changes, especially erosion, sclerosis and ankylosis. However, active inflammation of bone marrow or soft tissues is usually not recognized by standard CT. Therefore, a central part of the ASAS definition of active sacroiliitis and the classification of axSpA is inaccessible in CT exams [7]. In addition, CT involves ionizing radiation that is directly proportional to the volume of the patient in the X-ray beam. As patients with suspected axSpA are comparatively young, the risks associated with ionizing radiation are higher than in older populations. Furthermore, the SIJ are in spatial proximity to susceptible organs in the pelvic region, e.g. ovaries or testicles. All those factors contribute to the hesitancy of clinicians and radiologists to use CT for diagnosing axSpA.

## Low-Dose CT: Reducing Radiation Exposure

### What Is Low-Dose CT?

The radiation exposure in CT is dependent on multiple factors, some of them being the patient’s size and scan region (both determine the total volume), contrast of the objects in question and desired image quality and reconstruction methods [8]. While most radiologists agree that standard CT involves more radiation than a radiograph of the same body part, there is no definition of what constitutes a low-dose CT (ldCT) other than having comparatively lower radiation than a standard CT scan with the same anatomical coverage. Therefore, despite most scanners having established low-dose protocols implemented, the final radiation exposure of the patient will vary between vendors, institutions and machines [9].

### How to Achieve Low Dose?

In simple terms, the amount of CT radiation exposure influences the image noise. The higher the contrast of the pathology (e.g. erosion or syndesmophytes) to the surroundings, the more tolerable is an increase in noise and, therefore, low dose [10]. The cortical bone has especially high contrast to other structures, so evaluation of only the bone allows for lower radiation than soft tissues. Several other techniques can further reduce the exposure on the scanning side (e.g. volume scans, reduction of tube current or tin-filtration) or on the part of image reconstruction. For the latter, there have been important advances by using artificial intelligence for image reconstruction.

**Fig. 1:**
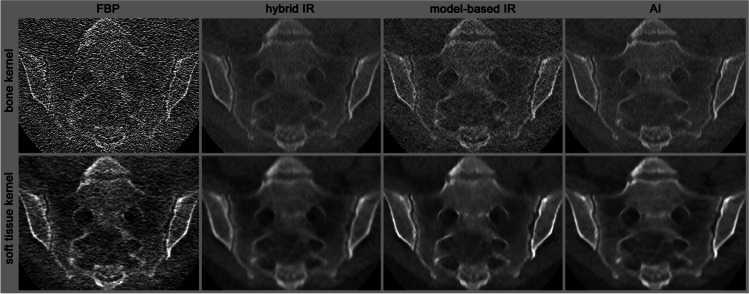
Different reconstructions of the same sacroiliac joint scan. This female patient underwent ultra-low-dose CT of the sacroiliac joints. The exposure of this scan was 0.07 mSv, comparable to a chest radiograph. The CT data were reconstructed in bone and soft-tissue kernel with filtered back projection (FBP), two versions of iterative reconstruction (IR) and artificial intelligence (AI)-based neural network. While the FBP images are non-diagnostic, the image quality sufficiently increases with the latest reconstruction techniques

Filtered back projection was the standard for CT reconstruction for years. However, advances in computer technology allowed the introduction of other techniques that decrease image noise and, thus, radiation exposure while maintaining sufficient image quality [11, 12]. The newest developments use artificial neural networks to achieve the same goal with even better results [13]. These techniques provide the opportunity to reduce radiation to the level of or even below radiography for specific applications (see Fig. 1).

### Low-Dose CT of the Sacroiliac Joints

**Fig. 2:**
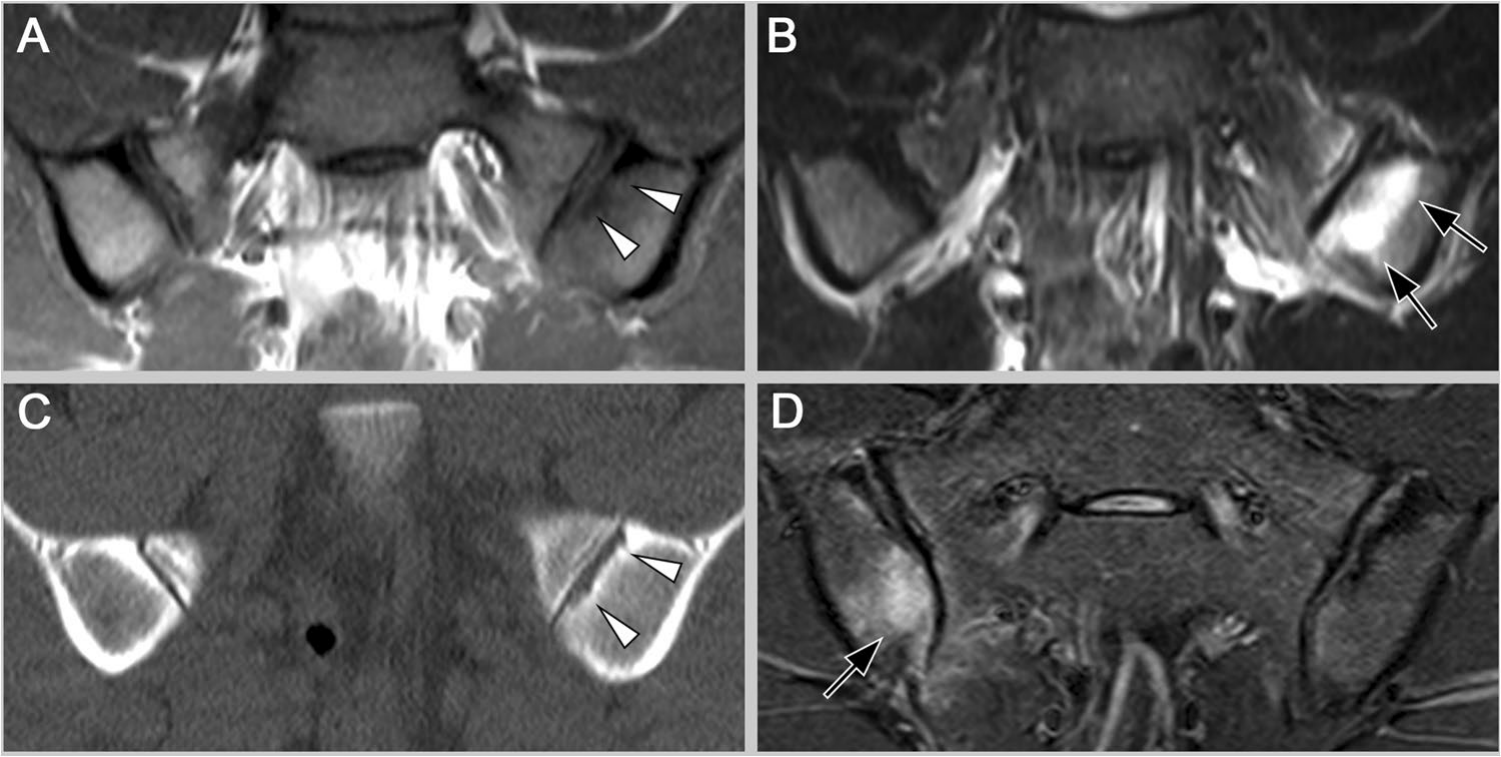
Mechanical stress or axSpA? This 28-year-old male athlete presented with inflammatory back pain. In the initial MRI, T1 (**A**) showed no unequivocal erosion (arrowheads). However, STIR (**B**) showed active inflammation in a region typical for mechanical stress reactions (arrows). With this uncertainty, the patient underwent ldCT (**C**) that confirmed erosion of the articular surface (arrowheads), and thus, the diagnosis of axSpA could be established. Follow-up MRI (**D**) after 2 years with new bone marrow oedema (arrow) further increased the confidence in this diagnosis

**Fig. 3:**
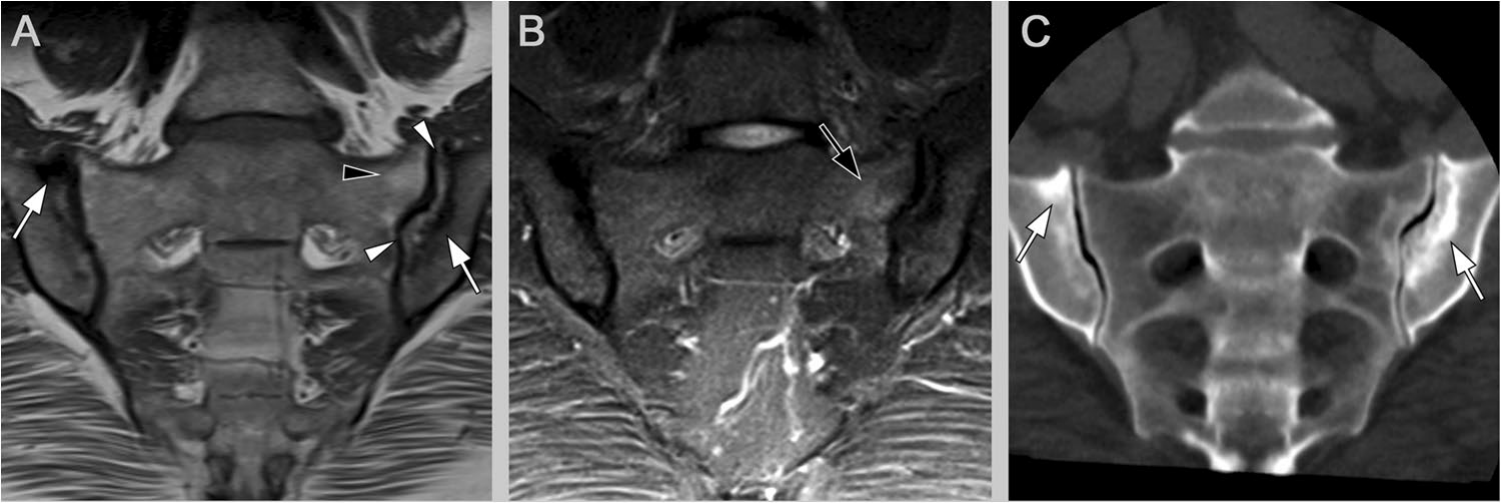
Degenerative joint disease. T1w MRI (**A**) in a 50-year-old female patient shows sclerosis (white arrows), fat metaplasia (black arrowhead) and fat metaplasia near the articular surface that appears very similar to backfill (fatty metaplasia inside an erosion) which is usually only seen in axSpA. There is also some bone marrow oedema in STIR (**B**, black arrow). CT (**C**), however, confirms sclerosis but no other structural lesions, especially no erosions. Therefore, and in accordance with clinical findings, the diagnosis of axSpA was refuted, and osteoarthritis/degenerative joint disease was confirmed

In daily practice, ldCT has established itself as a problem-solving tool for patients with unclear MR findings [14]. The strength of CT is to unambiguously prove or rule out the presence of structural lesions at the SIJ in regions that might be hard to assess on MRI when bone marrow lesions such as sclerosis or bone marrow oedema are nearby (see Fig. 2). Here, it shows higher sensitivity for erosion than radiography [15]. In this study by Ye et al., CT was less sensitive for structural lesions in axSpA patients than MRI (22% of MRI-positive axSpA patients were missed by CT although the authors did not specify which MRI lesions were missed on CT); however, a considerable proportion of the control population without a diagnosis of axSpA was MRI-positive for either bone marrow oedema (38%) or structural lesions (31%). In clinical practice, a 2022 published study found that CT had superior sensitivity for the diagnosis of axSpA compared to radiography (76 vs 66%) but low sensitivity compared to MRI (82%). On the other hand, CT had much higher specificity (97%) than radiography (68%) and MRI (86%) [16••] (see Fig. 3). Given a positive likelihood ratio of 28, ldCT was the only modality that could establish the diagnosis of axSpA to a sufficient degree in this analysis [17]. However, no negative imaging test was suitable to rule out the diagnosis.

The same group found that ankylosis and erosion were the most suitable imaging findings for diagnosis. However, erosions located in the ventral parts of the joint were less specific, and sclerosis did not prove helpful at all [18•]. Those definitions might not only be useful for clinical practice but also for training artificial intelligence programmes for sacroiliitis screening in CT scans performed for other reasons [19].

Apart from diagnostic considerations, CT remains the reference standard for structural lesions in several studies, especially those that aim at improving the diagnostic performance of MRI. One recent MRI trend is to generate CT-like images and thereby avoid radiation exposure by CT and to combine its superior depiction of erosion with MRI’s ability to detect bone marrow changes. Several techniques have recently been introduced, for example, zero echo time MRI [20], susceptibility-weighted imaging [21] or artificial intelligence based synthetic-CT [22•]. These studies all agree that CT is a valuable diagnostic modality.

### Low-Dose CT of the Spine

Only a minority of patients with axSpA show imaging changes restricted to the spine without findings in the SIJ; consequently, CT of the spine is usually used to assess late-stage complications and disease progression for clinical studies. ldCT has proven beneficial for detecting spinal trauma in a minor trauma setting compared to radiography [23] or standard CT [24]. Therefore, it can help detect spinal insufficiency, the so-called chalk-stick, fractures in patients with ankylosing spine disease [25].

**Fig. 4:**
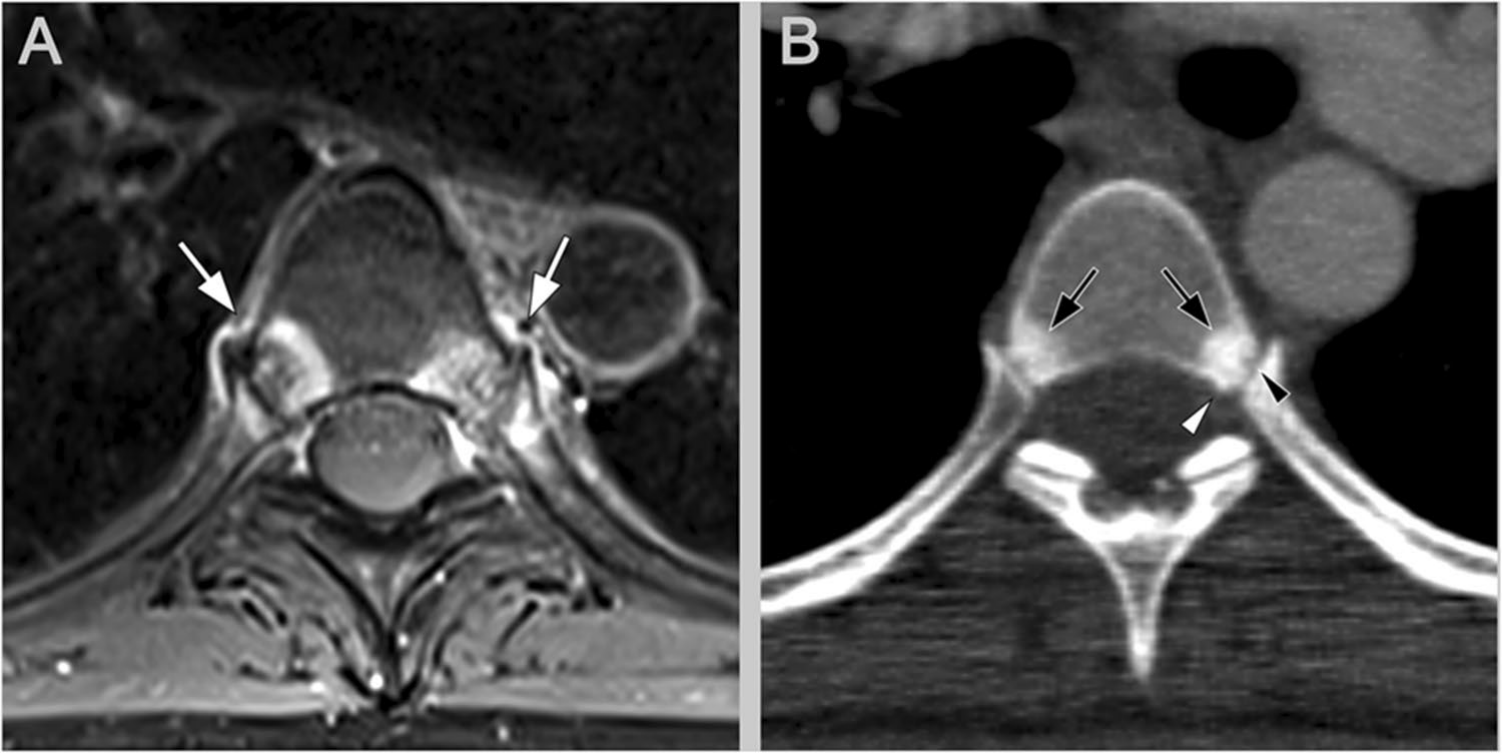
Costovertebral joint arthritis. This 37-year-old female patient with sacroiliitis and breath-dependent back pain has costovertebral joint arthritis on contrast-enhanced MRI (**A**, white arrows). CT (**B**) shows sclerosis (black arrows), erosion (black arrowhead) and periosteal proliferation (white arrowhead), typical for axSpA

In terms of lesion detection, CT showed a higher sensitivity for structural lesions and disease progression than radiography as it can assess the thoracic spine, which is usually omitted in radiography due to superposition of the ribs [26, 27]. Therefore, CT might reduce study time in clinical trials or a more detailed comparison of efficient drugs. CT’s superior spatial resolution also makes small joints of the spinal column accessible to evaluation that were previously not evaluable with radiography (Fig. 4). Recent studies analysed the facet joint as part of the posterior elements of the spine and found that they were also frequently affected in axSpA, especially in the thoracic spine. Therefore, adding facet joints would improve the detection of disease progression [28•] which may be important because facet joint involvement results in functional impairment [29].

## Dual-Energy CT: More Information

Another trend in CT imaging is spectral information with dual-energy CT (DECT) or similar approaches [30]. DECT increases the amount of information in a CT scan similar to colour photography compared to black–white films. While there are several applications of DECT in musculoskeletal imaging [31] including detection and characterization of gouty tophi [32, 33] and metal artefact reduction [34], the most important information for axSpA patients that can be derived from such scan is detection of bone marrow oedema [35•].

### Dozens of Techniques

There are several different techniques for acquiring spectral CT information that can either be generator- or detector-based. Those techniques have different advantages and disadvantages regarding the quality of the additional information and radiation exposure. However, it is beyond the scope of this article to explain them in detail. Nevertheless, the reader should keep in mind that techniques described in one article might not be transferable to their institution because of hard- and software restrictions, and therefore, their results in clinical practice might differ from what is described in the literature. The second point to consider is that the DECT method came into practice in the last few years and is still developing fast. For example, photon-counting detectors were introduced recently to clinical practice and promise DECT information from all scans with higher spatial resolution and less radiation but without increasing the radiation exposure [36]. It is possible that photon-counting or similar techniques will become the standard for DECT in the future and might be routinely available with most CT scans. It is an exciting prospect to see how this will develop in the next few years.

### Radiation Exposure Considerations in DECT

As described above, an essential point of low dose in CT is the high contrast of the objects of concern to surrounding tissue. While calcified bone structures themselves have high contrast to their surroundings, oedema compared to fatty bone marrow has much less. Additionally, the complex postprocessing of DECT images is susceptible to image noise. Therefore, DECT cannot currently be performed at low-dose levels. For example, the two available studies for sacroiliitis used a mean CT dose index of 7.4 and 20.9, respectively, which transfers to radiation exposure of approximately 2 to 5 mSv and is more in line with the dose for standard CT [35•, 37].

### Bone Marrow Oedema in axSpA

**Fig. 5:**
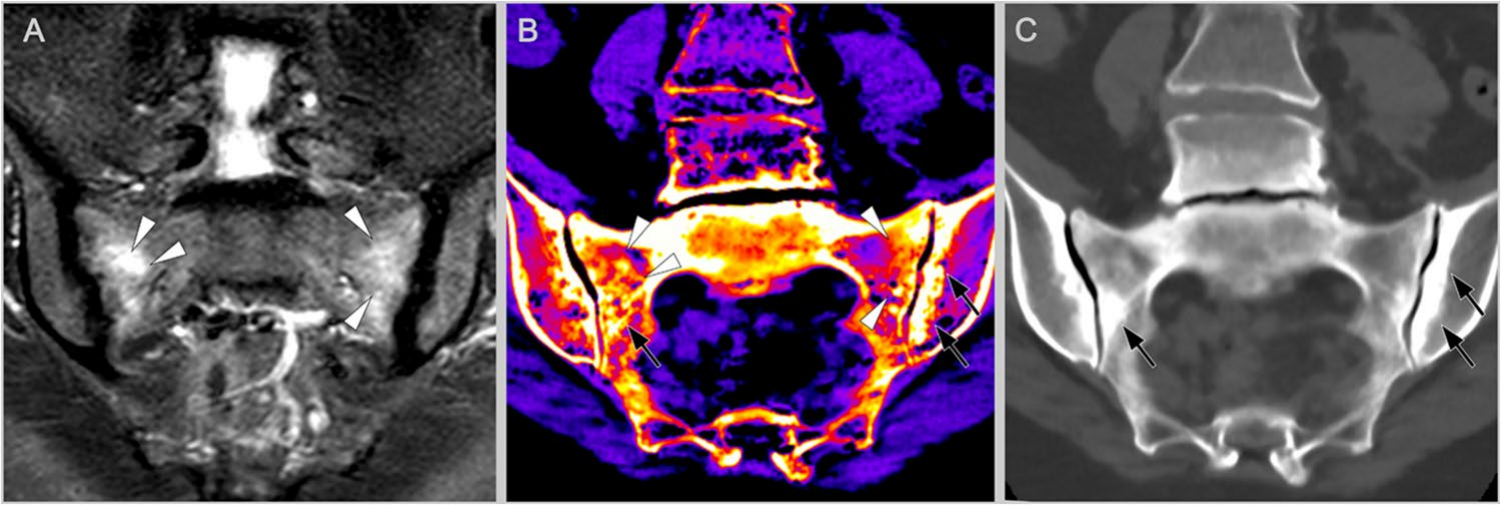
DECT of the sacroiliac joints in Osteitis condensans ilii. MRI-STIR (**A**), DECT virtual non-calcium maps (**B**) and conventional CT reconstructions (**C**) of a 59-year-old female patient with bilateral bone marrow oedema (arrowheads) and sclerosis (arrows) in Osteitis condensans ilii. In the bone marrow reconstructions (**B**), normal bone marrow is black to blue and oedema red to yellow. Especially on the left sacrum, it is hard to distinguish oedema and sclerosis. Therefore, a careful comparison of standard CT reconstructions and DECT maps is necessary, and results have to be interpreted with caution

Several dozens of manuscripts concern the application of DECT for detecting bone marrow oedema in a trauma setting [38] or osteoporosis [39], but data on arthritides, especially of the axial skeleton, is sparse. Currently, there are two studies on the subject reporting relatively high sensitivities (between 81 and 93%) and specificities (91 to 94%), respectively [35•, 37] using different DECT techniques and analysis methods. However, sclerosis seems to be a significant distracting factor deemed to reduce the diagnostic accuracy because it might be indistinguishable from oedema under certain circumstances (see Fig. 5). Moreover, there are several other limiting factors for clinical practice. For example, DECT bone marrow images are probably not as sensitive for small areas of oedema as MRI. Furthermore, the bone marrow composition in the peripheral skeleton is different from the axial skeleton, especially in younger patients because of the high proportion of red marrow in the axial skeleton. Erythropoietic marrow contains a more bound water that is indistinguishable from oedema on CT, but the two are easily distinguished on MRI. This results in a much higher contrast of bone marrow oedema on DECT at the hand [40, 41] than the SIJ or vertebral bodies. Probably, those are some reasons why studies of DECT for spine arthropathy have not yet been published. With the relatively high radiation dose needed to calculate the images, it is unclear whether DECT will ever become a standard imaging for axSpA patients. However, new results with photon-counting detectors must be awaited.

### Other Information from DECT

Despite the uncertainties with osteitis detection, there are several other possible advantages for axSpA patients undergoing DECT instead of conventional CT. For example, DECT is based on a similar principle as dual-energy X-ray absorptiometry (DEXA) and can derive quantitative measures for bone mineral density to predict osteoporotic fractures from spine or abdominal scans [42, 43]. In addition, other authors used bone marrow oedema reconstructions or collagen-sensitive maps to uncover disc pathologies such as herniations [44–46] or degeneration [47, 48], both giving valuable additional information in the context of back pain. Lastly, there are attempts to utilize the superior spatial resolution and the contrast sensitivity of DECT to detect active soft-tissue inflammation in the extremities, thus posing a quantifiable objective alternative to ultrasound and MRI [49]. The same is true for ldCT subtractions of the hand [50]. However, both techniques were, to date, not evaluated for the spine.

## Conclusion

CT is currently undergoing rapid development because of changes in computing power and artificial intelligence. Both ldCT and DECT have tremendous potential for imaging in rheumatology and for axSpA in particular. While the final clinical value of DECT is yet to be demonstrated, ldCT has been established as a specific imaging modality that might replace radiography and could be used as a meaningful supplement when MRI is not feasible or findings are ambiguous. Future developments in CT detector and reconstruction technology will further lower the radiation dose and improve resolution and its significance for clinical practice.
